# Comparison of treatment methods in patients with developmental dysplasia of the hip

**DOI:** 10.55730/1300-0144.5885

**Published:** 2024-07-12

**Authors:** Burhan KURTULUŞ

**Affiliations:** Department of Orthopedics and Traumatology, Ankara Dışkapı Yıldırım Beyazıt Training and Research Hospital,University of Health Sciences, Ankara, Turkiye

**Keywords:** Developmental dysplasia of the hip, Pemberton osteotomy, Salter innominate osteotomy, open reduction, closed reduction

## Abstract

**Background/aim:**

This study aimed to compare the results of Pemberton osteotomy (PO), Salter innominate osteotomy (SO), open reduction (OR), and closed reduction (CR) applied in the treatment of developmental dysplasia of the hip (DDH).

**Materials and methods:**

Included in the study were 101 hips of 82 patients treated at our orthopedic clinic between 2017 and 2023. The patients were evaluated preoperatively, postoperatively, and at the final follow-up. The results were evaluated based on Barret’s clinical and Severin’s radiological classifications. Those who developed avascular necrosis (AVN) were evaluated based on Bucholz–Ogden’s classification.

**Results:**

In terms of the preoperative acetabular angles (AAs), those for hips treated with PO were significantly higher than those of the other three, and those treated with SO were significantly higher than those of the other two (OR and CR) (p < 0.001). There was a significant difference in the final follow-up AAs of those treated with SO and PO compared to those treated with OR and CR (p < 0.001). The best corrections were achieved with PO (average: 27.94 ± 4.89°). There was a significant difference between PO and OR, and PO and CR in terms of the preoperative collodiaphyseal angles (CDAs) (p < 0.05). The greatest decrease was in those treated with PO (average: 22.44 ± 9.45°). AVN developed at various stages in 15 of 79 hips (14.85%) that were treated surgically. While AVN developed at a rate of 22.22% with PO, 18.18% with SO, and 17.85% with OR, no AVN developed in the 22 hips treated with CR.

**Conclusion:**

Understanding normal and abnormal values by age is essential for selecting appropriate treatments. Acetabulum-related surgeries should be planned for patients over 1.5 years of age with an AA above 30°. Early diagnosis and CR treatments yield excellent results and low AVN rates. Various DDH treatments in our clinic have shown low AVN rates, indicating safety and efficacy.

## 1. Introduction

Developmental dysplasia of the hip (DDH) includes a spectrum of hip developmental disorders that can present differently in different childhood age groups. The most common etiology is excessive looseness of the hip joint capsule and therefore, the inability to keep the femoral head within the acetabulum. As a result of this instability in the neonatal period, the femoral head may be partially (subluxated) or completely dislocated from the acetabulum [1].

Although the treatment for DDH varies depending on age, early diagnosis and initiation of treatment is very important for the prognosis of the disease. While it is possible to treat it with appropriate conservative methods in the early period, if the disease is not treated or it is overlooked, complicated surgical interventions may be needed in the future. If the appropriate treatment method is not chosen, conditions such as avascular necrosis (AVN) of the femoral head, insufficiency in acetabular coverage, and degenerative changes in the hip joint may occur [2–4].

Complete DDH treatment without leaving any residual deformity determines the patient’s quality of life in adulthood. The period in which the development potential of the hip joint is greatest is between 0 and 18 months. During this period, the hip joint responds very well to conservative methods. Unfortunately, this period is often skipped as a result of the lack of an adequate preventive medicine system and individuals, especially those living in rural areas, consulting a doctor too late. The majority of patients apply to hospitals after they begin walking [5–7].

The aim of DDH treatment is to provide an anatomically and functionally normal or near-normal hip joint. There are many treatment methods for this purpose at our clinic. The treatment method to be used is chosen according to the knowledge and experience of the orthopedist. Therefore, the goal in the treatment of DDH is to find and apply the most appropriate treatment option for each patient.

Hence, the aim of this study was to compare the results of Pemberton osteotomy (PO), Salter innominate osteotomy (SO), open reduction (OR) and closed reduction (CR) applied in the treatment of DDH.

## 2. Materials and methods

### 2.1. Patients and study design

Of the 284 patients treated at the Ankara Training and Research Hospital Orthopedics and Traumatology Clinic between January 2017 and January 2023, 82 patients who came for a checkup were included in the study. These patients were evaluated preoperatively, postoperatively, and at final follow-up according to the follow-up forms prepared by the researchers. General information of the patients, important points in their cases, physical examinations, radiological findings, and surgery-related findings were recorded on these forms. The preoperative hips of all the patients were evaluated by radiographs in the neutral anteroposterior (AP) and frog positions, and the postoperative hips were evaluated by AP radiographs in the standing, lying, and frog positions. In these radiographs, the acetabular angles (AAs) and collodiaphyseal angles (CDAs) were measured and recorded.

The patients were divided into 2 groups as Group 1: those treated surgically, and Group 2: those treated with CR. The patients treated surgically were divided into 3 groups as 1) those treated with SO, 2) those treated with PO, and 3) those treated with OR.

### 2.2. Evaluation

The patients’ results were evaluated according to Barret’s clinical and Severin’s radiological classifications [8,9]. Patients who developed AVN were evaluated according to Bucholz–Ogden’s classification [10]. Seventy-nine hips treated surgically were evaluated preoperatively according to Gage and Winter’s traction points [11].

#### 2.2.1. Barrett’s clinical classification

Group 1: (very good): Stable painless hip, no limping, Trendelenburg-negative, no limitation in movements.

Group 2: (good): Stable painless hip, slight limping, Trendelenburg-negative, slight limitation in movements.

Group 3: (moderate): Stable painless hip, slight limping, Trendelenburg-positive, moderate stiffness.

Bad: Unstable painful hip.

#### 2.2.2. Severin’s radiological classification

Group 1: (normal hip): Center-edge angle (CEA) >19° (for those aged 6–13 years), CEA >25° (for those over 14)

Group 2: Moderate deformity of the femoral head, neck or acetabulum, and other components of the joint are normal. CEAs of Groups 1a and 1b are the same.

Group 3: Dysplastic hip, but no sign of subluxation. CEA <15° (for those aged 6–13 years), CEA <20° (for patients over 14)

Group 4: (subluxation): Moderate degree (CEA is positive or zero), advanced degree (CEA is negative).

Group 5: Femoral head articulates with the false acetabulum above the acetabulum.

Group 6: (Redislocation).

### 2.3. Data analysis

The research data were analyzed using IBM SPSS Statistics for Windows 27.0 (IBM Corp., Armonk, NY, USA). Descriptive statistics such as the mean, standard deviation, maximum, minimum, and percentage were utilized to express the data distribution. Normality of the data distribution was assessed using the Kolmogorov–Smirnov test, and as the significance values were greater than 0.05, parametric tests were employed in advanced analyses. The independent sample t-test was used for two independent variables, One-way analysis of variance (ANOVA)was used for independent variables with more than two groups, and the Tukey honestly significant difference (HSD) test was used to determine the source of differences between groups. Statistical significance was accepted as p < 0.05.

## 3. Results

Treatment was administered to 101 hips of 82 patients. Surgical treatment was performed on 79 hips of 67 patients. Only CR and pelvipedal casting were applied to 22 hips of the remaining 15 patients. Of the patients, 72 were females (87.8%) and 10 were males (12.2%). The female:male ratio was 7.2:1. Of the 82 patients, 18 had bilateral DDH (21.95%), 34 had left DDH (41.46%), and 30 had right DDH (36.58%).

The ages of the patients were accepted as the age at which they started treatment. The ages of those treated surgically ranged from 12 to 50 months (average age: 25.3 months). Of these patients, the average age of those treated with SO was 26.4 ± 6.7 months, for those treated with PO it was 29.7 ± 8.3 months, and for those treated with OR it was 19.8 ± 6.0 months. The ages of the patients in Group 2 (treated with only CR and cast treatment) ranged between 8 and 19 (mean age: 11.8 ± 2.9) months.

There was no significant difference between the patients treated with SO and PO in terms of the average age of the 4 groups (p > 0.05). The age difference between the other groups was statistically significant (p < 0.05). Since the decision to opt for OR and CR treatment is made according to age, it is expected that there will be a significant difference between these treatment options and the age at which treatment begins, and that of patients treated with other surgical methods.

The average follow-up period for the patients was 25.14 months. The average follow-up period for those treated with PO was 26.32 months, for those treated with SO it was 27.24 months, and for those treated with OR it was 22.62 months. The average follow-up period for those treated with CR it was 24.38 months.

Of the patients, 5 were born via breech birth, 2 had low birth weights, 9 were born via cesarean section, and 73 were born via spontaneous vaginal delivery. Moreover, 22 patients had a family history of DDH.

Of the hips treated surgically, 16 had subluxation, 51 had dislocation, and 12 had high dislocation. Of the 22 hips treated with CR, 15 had dislocations and 7 had subluxations. There were none with high dislocation.

In the surgically treated group (79 hips), the lowest preoperative AA was 32° and the highest was 48° (mean: 38.5°). The mean preoperative AA was 43.5 ± 2.96° in those treated with PO and 38.6 ± 3.3° in those treated with SO. In those treated with OR, the mean AA was 33.4 ± 3.6°. The lowest CDA was 134° and the highest was 166° (mean: 154.4°). The average CDA of those treated with PO was 157.2 ± 6.4°, for those treated with SO it was 154.6 ± 6.6°, and for those treated with OR it was 151.4 ± 7.3°. Of the 22 hips treated with CR, the lowest AA was 24° and the highest was 34° (mean 28.55 ± 2.3), and the lowest CDA was 138° and the highest was 156° (mean 148.23 ± 5.7).

In the surgically treated group, the lowest final follow-up hip AA was 12° and the highest was 32° (average: 18.26°). The average was 15.56 ± 2.7° in those treated with PO, 16.85 ± 2.5° in those treated with SO, and 22.36 ± 4.1° in those treated with OR. The lowest CDA was 110° and highest was 151° (average: 136.24°). The mean CDA was 134.78 ± 8.2° in those treated with PO, 136.55 ± 9.3° in those treated with SO, and 137.46 ± 6.2° in those treated with OR. Of the patients treated with CR, the lowest final follow-up AA was 18° and the highest was 30° (mean: 21.36 ± 3.0), and the lowest CDA was 136°, while the highest was 150° (mean: 144.36 ± 4.6).

The preoperative and final follow-up AAs were compared for the 4 groups separately using the t test in paired groups (Table 1). The averages of the groups for the preoperative AAs were different than each other (p < 0.001). It was significantly higher in those treated with PO than in the other three treatments, and in those treated with SO, it was significantly higher than in the other two (OR and CR) (p < 0.05). That for OR was significantly higher than that for CR (p < 0.001).

There was no significant difference between SO and PO in the final follow-up AAs (p > 0.05). There was a significant difference between the final follow-up AAs of those treated with SO and PO compared to those treated with OR and CR (p < 0.001). There was no significant difference between OR and CR (p > 0.05). Additionally, when the 4 groups were compared separately, the final follow-up AAs were significantly reduced compared to the preoperative AAs (p < 0.001). There was a significant difference between the average correction degrees of the 4 groups (p < 0.001). The most correction was achieved with PO (mean: 27.94 ± 4.89°).

The preoperative and final follow-up AAs and CDAs were compared for the 4 groups separately in matched groups (Table 2). There was no significant difference between SO and PO, and SO and OR in terms of the preoperative CDAs (p > 0.05). There was a significant difference between PO and OR, and PO and CR (p < 0.05). The values for CR were significantly different between the other 3 groups (p < 0.05). There was a significant decrease in the differences between the preoperative and final follow-up CDAs in all 4 groups (p < 0.001).

Of the 79 hips of 67 patients treated surgically, 11 were treated with PO, four were treated with PO + derotation-variation osteotomy (DVO), three were treated with PO + DVO + shortening, 23 were treated with SO, seven were treated with SO + DVO, and three were treated with SO + DVO + shortening. However, 20 were treated with OR, two were treated with OR + DVO, two were treated with OR + DVO + shortening, and four were treated with only DVO. Of the 25 patients treated with DVO, a reconstruction plate was used for osteosynthesis in 14 and a limited contact dynamic compression plate (LC-DCP) plate was used in 11.

The cast immobilization period of the patients ranged from 45 days to three months (average: 1.85 months). Of the surgically treated hips, 69 (87.34%) were immobilized with a cast for two months postoperatively.

In the patients treated surgically, a Dennis–Brown splint was used for 35 to 70 days after the plaster cast (average: 55 days). In the patients treated with CR, a Dennis–Brown splint was used for 45 to 75 days (average: 62 days). Of the patients treated surgically, 54 were treated using the Smith–Petersen procedure, 21 were treated using the Smith–Petersen procedure + lateral incision, and four treated with only DVO underwent lateral incision. Adductor tenotomy was used in 54 (68.35%) of the hips treated surgically, and it was also used in 15 (68.18%) of the hips treated with CR.

According to the findings detected during intraoperative OR, the acetabulum was shallow in 52 (65.82%) hips. The teres ligament was intact and thickened in 62 (78.48%) hips. The ligamentum teres was ruptured in four (5.06%) and thinned in 13 (16.45%) hips. The limbus was inverted in 12 (15.18%) hips, everted in 46 (58.22%) hips, and normal in 21 (26.58%) hips. The posterior wall was inadequate in 18 (22.78%) hips. All patients treated with OR underwent iliopsoas tenotomy. They also all underwent careful capsulorrhaphy. Partial excision was performed in some of the hypertrophic capsules. If stabilization was deemed sufficient after OR, no additional intervention was performed.

The 79 hips treated surgically were evaluated preoperatively according to Gage and Winter’s traction points. Accordingly, 11 (61.11%) of the 18 hips treated with PO were −1 and 7 (38.88%) were 0. Of the 33 hips treated with SO, 17 (51.51%) were −1 and 16 (48.48%) were 0. Of the 28 hips treated with OR, 10 (35.71%) were evaluated as −1, 12 (42.85%) were evaluated as 0, and six (21.42%) were evaluated as 1.

When the 79 hips treated surgically were evaluated, 19 (57.57%) of the 33 treated with SO (seven were SO + DVO, three were SO + DVO + shortening) were classified as Barrett’s group 1 (very good), 11 (33.33%) were Barret’s group 2 (good), and three (9.09%) were Barret’s group 3 (fair) (90.9% good–very good).

Evaluation of the 101 treated hips based on Barrett’s clinical classification is shown in Table 3. All 22 hips (100%) treated with CR were classified as Barrett’s group 1 (very good). In total, 70 of the 101 hips (69.30%) were classified as Barret’s group 1 (very good), 26 (25.74%) were Barret’s group 2 (good), and five (4.95%) were Barret’s group 3 (middle) (overall: 95.14% good–very good) (Table 3).

Evaluation of the 101 treated hips based on Severin’s radiological classification is shown in Table 4. In total, 47 of the 101 hips (46.53%) were classified as Severin’s group 1, 37 (36.63%) were Severin’s group 2, 13 (12.87%) were Severin’s group 3, five (4.95%) were Severin’s group 4, and one (0.99%) was Severin’s group 5 (Table 4).

Comparison of the flexion values between the groups is shown in Table 5. During the postoperative follow-up, the hip movements of the patients were evaluated by dividing the patients into four groups, as those treated with SO (33 hips), those treated with PO (18 hips), those treated with OR (28 hips), and those treated with CR (22 hips). Hip flexion, abduction, and internal rotation movement values of the four groups at their final postoperative follow-up were evaluated statistically. There was a significant difference between the groups in terms of the hip flexion movements (p < 0.01). While there was no significant difference between the flexion values of those treated with SO, PO, and OR (p > 0.05), there was a significant difference between those treated with SO and PO and CR (p < 0.05) (Table 5).

Comparison of the abduction values between the groups is shown in Table 6. The same tests were applied for abduction. There was a significant difference between thefour groups based on the ANOVA (p < 0.05). In the Tukey HSD test, there was only a significant difference between those treated with PO and CR (p < 0.05). There was no significant difference between the other groups (p > 0.05).

Comparison of the internal rotation values between the groups is shown in Table 7. There was a significant difference between the four groups based on the ANOVA (p < 0.001). In the Tukey HSD test, there was no significant difference between those treated with SO and only PO (p > 0.05). There was a significant difference between the other groups (p < 0.05) (Table 7).

### 3.1. Evaluation of AVN

AVN at various stages developed in 15 of the 79 hips (14.85%) treated surgically. Of these, 9 (11.39%) had type 1 AVN according to Bucholz–Ogden’s classification, one (1.26%) had type 2 AVN, four (5.06%) had type 3 AVN, and one (1.26%) had type 4 AVN. AVN was present in four of the 18 hips (22.22%) treated with PO (three of which were type 1, and one was type 3). AVN was present in six of the 33 hips (18.18%) treated with SO (three were type 1, two were type 3, and one was type 4). AVN was detected in five of the 28 hips (17.85%) treated with OR (three were type 1, one was type 2, and one was type 3). AVN was not observed in the 22 hips treated with CR (Figure).

## 4. Discussion

The most significant finding in this study was that PO achieved the most substantial corrections in the preoperative AAs and CDAs, with an average AA correction of 27.94 ± 4.89° and a CDA decrease of 22.44 ± 9.45°. Additionally, patients treated with PO had the highest preoperative AAs compared to the other surgical methods (SO, OR, and CR) (p < 0.001). Furthermore, the development of AVN was observed in 22.22% of the hips treated with PO, 18.18% treated with SO, 17.85% treated with OR, and 0% treated with CR.

In this study, among the 67 patients treated surgically, the average age of those treated with PO was 29.72 ± 8.32 months, for those treated with SO it was 26.39 ± 6.75 months, for those treated with OR it was 19.79 ± 5.98 months, and for those treated with CR + cast treatment it was 11.8 months. None of these patients had previously received conservative treatment. Patients generally applied to our clinic for reasons such as a delay in walking, limping, and limitation of movement. In the statistical evaluation, there was no statistically significant difference between the ages of the patients treated with SO and PO. The patients treated with SO and PO were significantly older than those treated with OR and CR. The patients treated with OR were significantly older than those treated with CR. In the literature, it is recommended that SO and PO be performed after 18 months of age [12,13]. While SO can be performed in subluxated hips until adolescence, PO is limited to the age of 6 when the triradiate cartilage closes [14]. Since the oldest patient in the current study was 50 months old, there was no difference in the age at which they started treatment. Apart from this, the ages of the patients treated with OR and CR were consistent with the literature [12–14].

AVN is the most important complication of DDH treatment. Most researchers consider the change in blood flow to the femoral head to cause this iatrogenic complication. Vascular insufficiency in the proximal part of the femur after treatment covers a wide spectrum, ranging from mild to severe, and it has varying levels of impact on prognosis [15,16]. Traction before reduction, gentle manipulations, femoral shortening when necessary, and casting in the human position reduce the incidence of AVN [17–20]. In the present study, varying degrees of AVN were detected in the 15 patients treated (14.85%). This rate is low compared to the literature.

DDH is more common in the first child. The reason for this has been shown to be the low tension of the uterus and the tightness of the abdominal muscles [21]. Of the 82 patients treated herein, 44 (53.65%) were born as the first child, 28 (34.14%) were born as the second child, and the other 20 (24.39%) were born as the third and fourth children. These results are also compatible with the literature.

Studies conducted in families with DDH have shown that there is a genetic transmission in these patients [22,23]. DDH is 6–8 times more common in females than in males and was previously attributed to hormonal joint laxity [24]. However, it has been stated that joint laxity is genetically inherited [25]. Of the patients in the current study, 22 (26.82%) had a family history of DDH. However, the female/male ratio in these patients was 7.2.

In studies conducted during the neonatal period, right side involvement was reported to be less than the left in all patients diagnosed with DDH. This is explained by the position of the fetus in the womb during intrauterine life [26]. Of the patients in the present study, 34 (41.46%) had left side, 30 (36.58%) had right side, and 18 (22.5%) had bilateral DDH. The left:right ratio was low compared to the literature.

The practice of swaddling still continues, especially in rural areas. With the application of swaddling in the newborn period, the hips are forced into adduction and extension. Thus, lax hips create a basis for dislocation. Many researchers have stated that the majority of patients with joint laxity in the neonatal period recover spontaneously. Swaddling causes dislocation of these hips [27–30].

It is recommended that CR in the treatment of DDH be performed up to the age of three [31]. For the success of CR, the indication must be well established. It should not be forgotten that forced CRs may cause redislocation or AVN [32,33]. Herein, the average age of the 15 patients treated with CR was 11.8 months. When the results of the 22 hips were evaluated according to Barret’s clinical and Sever’s radiological classifications, excellent results were found. This was attributed to the fact that all the patients were under 1.5 years of age and the acetabulum was in the period when its development potential was at its highest.

In cases with mild acetabular dysplasia, adequate centralization of the femoral head to the acetabulum is achieved and adequate hip development is expected with OR and DVO under the age of three [34]. In some studies, it has been deemed appropriate to perform acetabulum-oriented surgeries in DDH between the ages of 1.5–3 years if the AA is 1.5 times that of their age group [35]. If the AA is 38° or above, PO is recommended. The reason for this is that an average of 10° (8–12°) correction can be achieved with SO. It was reported that the spontaneous development capacity of the salter acetabulum is maximum in the first 18 months and slows down significantly after that [36,37]. In the current study, the AA correction rates of all the patients divided into four groups were statistically significant. Accordingly, the greatest improvement was in the hips treated with PO (27.94 ± 4.89), followed by SO (21.76 ± 3.86), OR (11.04 ± 3.86), and CR (7.18 ± 2.08).

Although acetabular development is generally very rapid until the age of three, all researchers have stated that the potential for acetabular development continues, although slowly, until the age of 8–9 [38,39]. The preoperative and final follow-up CDAs in the current study were compared statistically. Accordingly, the decrease in the CDAs in patients treated with CR was significantly less than in those treated surgically. There was no statistically significant difference in the CDA recovery rates between the surgical methods. This result was attributed to the application of DVO to 25 of the surgically treated hips.

In some studies, intertrochanteric osteotomy and angled plate fixation are advocated in the treatment of DDH [40,41]. One study also reported using flat plates [42]. Nowadays, the use of angled plates is increasing due to the advantages of intertrochanteric osteotomy [40,41]. In the present study, femoral osteotomy was performed on seven (38.88%) of the hips treated with PO, 10 (30.3%) of the hips treated with SO, and eight (28.57%) of the hips treated with OR. Preoperative anteversion measurements and shortness measurements can be performed on patients who will undergo femoral osteotomy. It is evaluated whether it is in valgus or varus. However, intraoperative evaluation is more important. Herein, after completing surgical intervention for the acetabulum, the hip was reduced and femoral osteotomy was performed if deemed necessary. Femoral osteotomy was not performed on hips that had excessive preoperative anteversion and were stable with surgical intervention aimed only at the acetabulum. In all 25 hips treated with femoral osteotomy, osteotomy was performed from the subtrochanteric region, and a 4–5-hole reconstruction plate or LC-DCP plate was used. No delayed union or nonunion developed in any of the patients.

### 4.1. Limitation of the study

There were some limitations in the study. The fact that it was a single-center and retrospective study limited the comprehensiveness of the data due to the nature of the study. Additionally, the small sample size further restricted the generalizability of the findings.

## 5. Conclusion

Postoperative hip flexion, abduction, and internal rotation degrees of patients in four groups were statistically evaluated. The flexion values of the hips treated with CR were significantly higher than those of the hips treated with SO and PO. There was only a significant difference between PO and CR in terms of the abduction values. In terms of the internal rotation values, that for CR was significantly higher than the other three groups, and there was a significant difference between SO and PO. Therefore, the best hip movements were found in hips treated with CR.

In conclusion, the normal and abnormal AAs of the patients according to their ages should be well known and treatment methods should be carefully selected accordingly. Acetabulum-related surgeries should be planned for patients over 1.5 years of age with an AA above 30°. CR can provide excellent results as it is applied to patients at an early age. This reveals the importance of early diagnosis and early treatment in DDH patients. Many different treatment methods for DDH are successfully applied in our clinic, and the AVN rates were low in conservative treatment methods applied at an early age, and these treatment methods were found to be safe in terms of AVN. It has been observed that AVN rates after different surgical treatments are either consistent with the literature or low. In this respect, the age of treatment in DDH, the selection of appropriate treatment according to age, and the application of treatment methods with the appropriate technique are very important in terms of femoral head AVN.

## Figures and Tables

**Figure:**
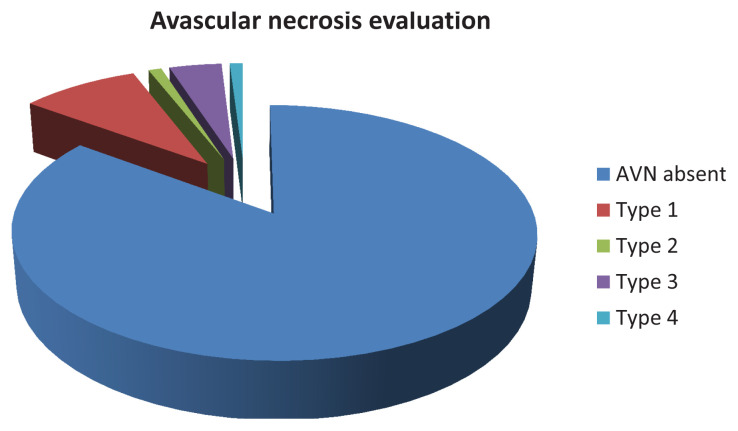
AVN evaluation of 101 treated hips according to Bucholz–Ogden’s classification.

**Table 1:** Comparison of mean acetabular angle (AA) values according to preoperative and final follow-up.

Tukey HSD			Mean difference	p-value
Preoperative AA	SO	PO	−4.89*	0.000
		OR	5.21*	0.000
		CR	10.06*	0.000
	PO	SO	4.89*	0.000
		OR	10.11*	0.000
		CR	14.95*	0.000
	OR	SO	−5.21*	0.000
		PO	−10.11*	0.000
		CR	4.85*	0.000
	CR	SO	−10.06*	0.000
		PO	−14.95*	0.000
		OR	−4.85*	0.000
Final follow-up AA	SO	PO	1.29	0.502
		OR	−5.51*	0.000
		CR	−4.52*	0.000
	PO	SO	−1.29	0.502
		OR	−6.80*	0.000
		CR	−5.81*	0.000
	OR	SO	5.51*	0.000
		PO	6.80*	0.000
		CR	0.99	0.686
	CR	SO	4.52*	0.000
		PO	5.81*	0.000
		OR	−0.99	0.686

* Statistically significant.

AA: acetabular angle; SO: Salter innominate osteotomy; PO: Pemberton osteotomy; OR: open reduction; CR: closed reduction; HSD: honestly significant difference.

**Table 2:** Comparison of collodiaphyseal angle (CDA) values according to preoperative and final follow-up.

Tukey HSD			Mean difference	p-value
Preoperative CDA	SO	PO	−2.62	0.531
		OR	3.21	0.236
		CR	6.38*	0.004
	PO	SO	2.62	0.531
		OR	5.83*	0.022
		CR	8.99*	0.000
	OR	SO	−3.21	0.236
		PO	−5.83*	0.022
		CR	3.17	0.337
	CR	SO	−6.38*	0.004
		PO	−8.99*	0.000
		OR	−3.17	0.337
Final follow-up CDA	SO	PO	1.77	0.852
		OR	−0.92	0.964
		CR	−7.82*	0.001
	PO	SO	−1.77	0.852
		OR	−2.69	0.636
		CR	−9.59*	0.001
	OR	SO	0.92	0.964
		PO	2.69	0.636
		CR	−6.90*	0.009
	CR	SO	7.82*	0.001
		PO	9.59*	0.001
		OR	6.90*	0.009

* Statistically significant.

CDA: collodiaphyseal angle; SO: Salter innominate osteotomy; PO: Pemberton osteotomy; OR: open reduction; CR: closed reduction; HSD: honestly significant difference.

**Table 3:** Clinical evaluation of 101 treated hips with Barrett’s.

	Barrett’s clinical evaluation			
Treatment types	Group 1	Group2	Group 3	Total
Salter osteotomy	19	11	3	33
Pemberton osteotomy	11	6	1	18
Open reduction	18	9	1	28
Closed reduction	22	-	-	22
Total	70	26	5	101

**Table 4:** Severin’s radiological evaluation of 101 treated hips.

	Severin’s radiological evaluation					
Treatment types	Group 1	Group2	Group 3	Group 4	Group 5	Total
Salter osteotomy	13	12	5	2	1	33
Pemberton osteotomy	6	7	4	1	-	18
Open reduction	12	10	4	2	-	28
Closed reduction	15	7	-	-	-	22
Total	47	37	13	5	1	101

**Table 5:** Comparison of flexion values between groups.

Tukey HSD		Mean difference	p-value
Salter osteotomy (SO)	PO	3.99	0.568
	OR	−2.12	0.861
	CR	−8.03*	0.033
Pemberton osteotomy (PO)	SO	−3.99	0.568
	OR	−6.11	0.224
	CR	−12.02*	0.003
Open reduction (OR)	SO	2.12	0.861
	PO	6.11	0.224
	CR	−5.91	0.205
Closed reduction (CR)	SO	8.03*	0.033
	PO	12.02*	0.003
	OR	5.91	0.205

* Statistically significant.

SO: Salter innominate osteotomy; PO: Pemberton osteotomy; OR: open reduction; CR: closed reduction; HSD: honestly significant difference.

**Table 6:** Comparison of abduction values between groups.

Tukey HSD		Mean difference	p-value
Salter osteotomy (SO)	PO	1.97	0.693
	OR	−1.96	0.601
	CR	−3.94	0,097
Pemberton osteotomy (PO)	SO	−1.97	0.693
	OR	−3.93	0.154
	CR	−5.91*	0.016
Open reduction (OR)	SO	1.96	0.601
	PO	3.93	0.154
	CR	−1.98	0.670
Closed reduction (CR)	SO	3.94	0.097
	PO	5.91*	0.016
	OR	1.98	0.670

* Statistically significant.

SO: Salter innominate osteotomy; PO: Pemberton osteotomy; OR: open reduction; CR: closed reduction; HSD: honestly significant difference.

**Table 7:** Comparison of internal rotation values between groups.

Tukey HSD		Mean difference	p-value
Salter osteotomy (SO)	PO	3.91	0.133
	OR	−4.10*	0.049
	CR	−8.94*	0.000
Pemberton osteotomy (PO)	SO	−3.91	0.133
	OR	−8.02*	0.000
	CR	−12.85*	0.000
Open reduction (OR)	SO	4.10*	0.049
	PO	8.02*	0.000
	CR	−4.84*	0.032
Closed reduction (CR)	SO	8.94*	0.000
	PO	12.85*	0.000
	OR	4.84*	0.032

* Statistically significant.

SO: Salter innominate osteotomy; PO: Pemberton osteotomy; OR: open reduction; CR: closed reduction; HSD: honestly significant difference.
